# Low isoniazid and rifampicin concentrations in TB/HIV co-infected patients in Uganda

**DOI:** 10.7448/IAS.17.4.19585

**Published:** 2014-11-02

**Authors:** Christine Sekaggya Wiltshire, Mohammed Lamorde, Alexandra Scherrer, Joseph Musaazi, Natascia Corti, Buzibye Allan, Rita Nakijoba, Damalie Nalwanga, Lars Henning, Amrei Von Braun, Solome Okware, Barbara Castelnuovo, Andrew Kambugu, Jan Fehr

**Affiliations:** 1 Infectious Diseases Institute, Research, Kampala, Uganda; 2 Infectious Disease and Hospital Epidemiology, University Hospital of Zurich, Zurich, Switzerland; 3 Clinical Pharmacology and Toxicology, University Hospital of Zurich, Zurich, Switzerland

## Abstract

**Introduction:**

There is limited data available on exposure to anti-tuberculosis (TB) drugs in this region. Peloquin has described reference ranges [1] however some studies have demonstrated that patients actually achieve concentrations below these ranges [2]. There is limited data about exposure to anti-TB drugs in the HIV/TB co-infected population in Sub-Saharan Africa. Our objective is to describe the concentration of anti-TB drug levels in a well characterized prospective cohort of adult patients starting treatment for pulmonary TB.

**Methods:**

This study is an ongoing study carried out in the TB/HIV integrated clinic at the Infectious Diseases Institute in Kampala, Uganda. Sputum culture and microscopy was done for all patients. We performed pharmacokinetic blood sampling of anti-TB drugs for 1 hour, 2 hours and 4 hours post dose at 2 weeks, 8 weeks and 24 weeks after initiation of anti-TB treatment using ultraviolet high-performance liquid chromatography (UV-HPLC). We described the maximum concentration (Cmax) of isoniazid (H), rifampicin (R), ethambutol (E) and pyrazinamide (Z) and compare them with the values observed by Peloquin et al. referenced in other studies.

**Results:**

We started 113 HIV infected adults on a fixed dose combination of HREZ. The median age of our population was 33 years, of which 52% were male with a median BMI of 19 kg/m^2^ and a median CD4 cell count of 142 cells/µL. In 90% of the participants, the diagnosis of TB was based on microscopy and or cultures. The boxplot graph shows the median Cmax and IQR of H and R.

Levels of H were found to be below the reference ranges (3–6 µg/mL) in 54/77(70.1%), 38/59(64.4%) and 15/24(62.5%) participants at weeks 2, 8 and 24. Rif levels were also found to be below the reference ranges (8–24 µg/mL) in 41/66(62.1%), 26/48(54.2%) and 8/10(8%) participants at weeks 2, 8 and 24, respectively. The mean Cmax of E and Z were within the reference range at week 2 and 8; mean Cmax of 3.2±SD2.1 µg/mL and 4.0±SD3.1 µg/mL for E and 41.6±SD13.1 µg/mL and 42.6±SD16.4 µg/mL for Z.

**Conclusion:**

We observed lower concentrations of isoniazid and rifampicin in our study population of HIV/TB co-infected patients. The implications of these findings are not yet clear. We therefore need to correlate our findings with the response to TB treatment.

**Figure 1:**
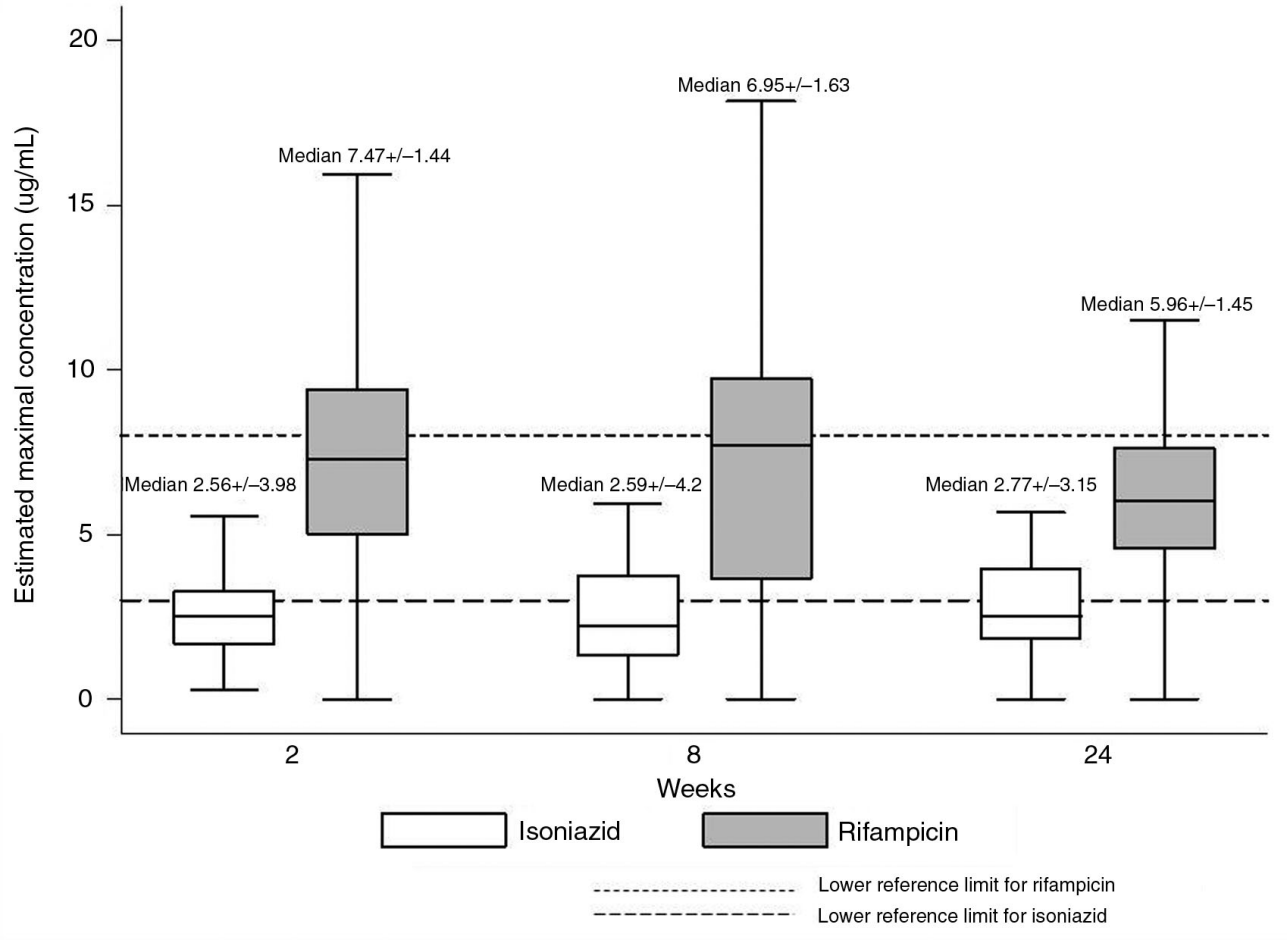
Maximum drug concentrations in comparision to reference ranges.
